# Metabolic and Lactation Effects of Rumen-Protected Choline Supplementation in Peripartum Dairy Cows and Its Effects on Calf Growth Until Weaning

**DOI:** 10.3390/metabo16020122

**Published:** 2026-02-10

**Authors:** Ugur Serbester, Melisa Topaktas

**Affiliations:** Department of Animal Science, Çukurova University, 01330 Adana, Türkiye; yesilbudakmelisa@gmail.com

**Keywords:** choline, metabolic parameters, dry matter intake, calf performance

## Abstract

Background: This study evaluated the effects of rumen-protected choline (RUPCHOL) supplementation in dairy cows from 21 days before calving to 28 days postpartum. The objective was to determine how RUPCHOL influences metabolic status, milk composition, and subsequent calf growth until weaning. Methods: Twenty-seven pregnant Holstein cows were assigned to a Control group (*n* = 13) or an RUPCHOL group (*n* = 14), both receiving a total mixed ration (TMR), with the RUPCHOL group supplemented with 15 g/day of choline chloride. Cows were monitored during prepartum, calving, and postpartum periods for body weight, body condition score, dry matter intake, rectal temperature, milk yield and composition, and blood metabolites. Results: RUPCHOL supplementation tended to reduce serum aspartate aminotransferase and lowered concentrations of non-esterified fatty acids and β-hydroxybutyrate, indicating improved metabolic status. Milk total solids, fat, and protein percentages were higher in RUPCHOL-fed cows, suggesting enhanced milk quality. Maternal supplementation did not affect colostrum immunoglobulin G (IgG) content or calf body weight and body measurements (heart girth, wither height, hip height, and body length) from birth to weaning. Conclusions: In summary, RUPCHOL supplementation improved indicators of metabolic health and milk composition of dairy cows during the peripartum period without altering calf growth outcomes.

## 1. Introduction

The peripartum, or transition period, is defined as the time from three weeks before calving to three to five weeks after calving [1,2]. During this period, dairy cows undergo significant physiological and metabolic adaptations. These changes are marked by low dry matter intake (DMI) and a rapid rise in nutrient requirements for survival, pregnancy, and milk production [3]. These conditions lead to a negative energy balance, necessitating body fat mobilization to meet nutrient demands. Consequently, blood non-esterified fatty acid (NEFA) concentrations increase during the transition period. NEFA can be used for milk production or processed by the liver and released into the bloodstream as very-low-density lipoprotein (VLDL). However, excessive NEFA transport to the liver may cause triglyceride accumulation, leading to metabolic disorders such as fatty liver and ketosis. Additionally, the physiological and metabolic changes during pregnancy can alter intrauterine conditions and may influence the lifelong performance and survival of offspring [4]. Altered nutrient availability and metabolic stress in the dam may affect organ development, immune function, and nutrient absorption in the neonate, thereby influencing calf growth and overall health. These prenatal adaptations can significantly impact performance until weaning. For instance, offspring from cows experiencing high metabolic stress may exhibit compromised growth and immune responses. This results in lower weight gain and increased illness susceptibility during pre-weaning [4]. Recent studies have demonstrated that nutritional interventions can modulate immune function and gut integrity in livestock, which may be particularly relevant during periods of metabolic stress [5,6].

Various nutritional and management strategies have been developed to support the health and welfare of both dam and fetus during the transition period. One approach is supplementing maternal diets with nutrients that may be limited. Given the typical increase in adipose tissue mobilization and hepatic lipid synthesis after calving, lipotropic agents like choline have been investigated for their potential to improve health and performance [7,8]. Choline’s role in transmethylation reactions, which are essential for metabolism, gene expression, and one-carbon metabolism, has further increased interest in its supplementation. Furthermore, choline and related metabolites have been shown to influence inflammatory responses and lipid metabolism pathways in various mammalian species [9,10], suggesting potential benefits beyond traditional lipotropic effects.

Research on rumen-protected choline (RUPCHOL) has primarily focused on peripartum cows, with limited and variable data on its effects during later lactation stages. This study investigated the effects of RUPCHOL supplementation from 21 days before calving to 28 days postpartum on dairy cow performance, milk composition, blood metabolic parameters, rectal temperature, and total milk yield at 150 days. Additionally, we examined the impact of maternal RUPCHOL on colostrum IgG levels and composition (total solids, fat, protein, casein, lactose, and urea-N). We also assessed calf body weight and body measurements (heart girth, wither height, hip height, and body length) from birth to weaning.

## 2. Materials and Methods

### 2.1. Ethical Approval, Study Location, and Conditions

Approval for the procedures performed on the cows used in this study was obtained from the Çukurova University Health Sciences Experimental Application and Research Center (Meeting number and date: 8 and 16 December 2022). The research was conducted at the Cukurova University Research and Application Farm Dairy Cow Unit between October and December 2022. The environmental conditions during the experiment were as follows: temperature: 19.0 ± 2.86 °C, humidity: 62.0 ± 13.64%, and temperature–humidity index: 64.3 ± 3.93.

### 2.2. Animal Material, Experiment Duration, and Housing

#### 2.2.1. Dams

Thirty Holstein pregnant cows were used in the study. The cows were housed in individual pens from approximately 21 days prepartum until 28 days postpartum, after which they were returned to the production herd. During the study, cows were kept in concrete floor pens with individual waterers and feeders, and wheat straws were used as litter material.

#### 2.2.2. Experimental Design

The cows were randomly allocated into two groups (Control and RUPCHOL) based on body weight, body condition score, and lactation number to ensure a balanced distribution of animals across treatments. In the Control group, mean (± standard deviation) body weight was 618.2 ± 80.58 kg, body condition score (BCS) was 3.6 ± 0.71, and lactation number was 2.4 ± 0.89. In the RUPCHOL group, mean (± standard deviation) body weight was 627.6 ± 101.81 kg, BCS was 3.8 ± 0.65, and lactation number was 2.6 ± 0.49. All animals were multiparous dry cows in the late gestation period; no pregnant heifers were included.

The treatment group received a microencapsulated choline premix (Ruprocol^®^, Vetagro, Reggio Emilia, Italy) containing 25% choline chloride, with hydrogenated palm oil as the carrier. Following the manufacturer’s recommendation, 60 g of RUPCHOL was top-dressed onto each cow’s individual TMR during morning feeding. This provided 15 g choline chloride per cow daily (60 g RUPCHOL × 25% choline chloride). Based on the molecular weights of choline chloride (139.62 g/mol) and choline ion (104.17 g/mol), the 15 g/day choline chloride provided approximately 11.2 g/day of choline ion (15 × 104.17/139.62 = 11.2 g/day). Cows consumed the entire supplemented TMR portion, ensuring complete intake of the added RUPCHOL. This rumen-protected choline formulation is estimated to allow for 60–80% of the choline in the product to escape ruminal degradation [11,12], whereas almost all unprotected choline would be degraded in the rumen [13].

#### 2.2.3. Feed Material and Feeding Applications

Cows were fed a total mixed ration (TMR) consisting of concentrate and roughage (corn silage, lucerne hay, and wheat straw). The TMRs were formulated to meet or exceed the nutritional requirements of late-gestation dry cows (approximately 620–630 kg body weight) and early lactation cows (around 630 kg body weight) according to NRC [14] recommendations (Table 1). The lactation ration was balanced to support an expected milk yield of approximately 30 kg/day containing 4% fat and 3.2% protein. The roughage-to-concentrate ratio in the TMR was 75:25 during the dry period and 60:40 during lactation.

#### 2.2.4. Calves

Cows showing signs of labor pain (swelling of the vulva, loosening of pelvic ligaments, vaginal discharge, etc.) were moved to the maternity area. Calves were housed with their dams for 72–96 h postpartum and were ensured to receive colostrum at least twice a day before being moved to individual polyethylene hutches. Calves were fed 5 L of whole milk daily, divided equally between morning and evening feedings. Starting from the 2nd week, calves were given lucerne hay and calf starter feed ad libitum.

### 2.3. Data Collection

#### 2.3.1. Dam Measurements

Individual body weights were recorded weekly on an electronic scale after morning milking and before feeding. Body condition score was assessed visually by a trained observer using the 5-point system with 0.25-unit increments as described by Ferguson et al. [16]. Daily DMI was calculated by subtracting the feed given from the feed not consumed. Rectal temperatures were measured weekly with a digital thermometer inserted 10–11 cm into the rectum and touching the rectal wall during body weight weighing.

After calving, the dams and calves were housed together for 72–96 h, the calves were separated, and the dams were moved to the experimental pens used in prepartum and milked twice in the central milking system in the morning (04:00) and evening (16:00). Cows that completed the 29th postpartum day were transferred to the production herd. Daily individual milk yields of the cows (from postpartum day 3 or 4 to day 150) were obtained from the milking system (DelPro, Version 5.3, De Laval, Tumba, Sweden).

In order to determine individual milk compositions, milk samples were collected from morning milking on postpartum days 7, 14, 21, and 28 with the help of sample collection cups attached to the milking system. The milk samples were firstly counted for somatic cell count (CellCount, DeLaval, Sweden) and then analyzed for total solids, fat, protein, lactose, casein, and urea using a Fourier Transform Infrared Spectroscopy-based milk analyzer (FT-120, Milkoscan, FOSS, Hillerød, Denmark). Milk urea nitrogen levels (mg/dL) were calculated by multiplying the analysis result by 466.7 ([(atomic weight of N/atomic weight of urea molecule) × 1000]). Fat and protein yields were calculated by multiplying these variables by milk yield. The following equation was used to calculate milk yield adjusted for 4% fat corrected milk yield [17].

4% fat corrected milk yield (kg/d) = [Milk yield (kg/d) × (0.4 + 0.15 × Milk fat (%)](1)

Blood samples were collected prepartum (days −21, −14, and −7), at calving, and postpartum (days 7, 14, and 21). Samples were collected into two types of vacuum tubes: plain tubes without anticoagulant for serum separation, and heparinized tubes for plasma separation. Blood samples in plain tubes were allowed to clot at room temperature for 30 min, then centrifuged at 3000× *g* for 15 min to obtain serum. Blood samples in heparinized tubes were immediately centrifuged at 3000× *g* for 15 min to obtain plasma. Serum was used for biochemical analysis (glucose, Ca, Mg, P, total protein, BUN, albumin, AST, GGT, and cholesterol) using a biochemistry analyzer (BS-300, Mindray, Shenzhen, China) and instrument-specific kits (Bioanalytic Diagnostic Industry, Hamburg, Germany) in a private laboratory. Plasma NEFA and BHB concentrations were determined by the ACS-ACOD method (kit NEFA-C, Wako Chemicals, Neuss, Germany) and the d-3-hydroxybutyrate kit (Randox Laboratories Ltd., Ardmore, UK), respectively.

#### 2.3.2. Calves Measurements

Colostrum samples were collected immediately at calving, with an additional sample taken 36 h postpartum to assess transition milk. IgG concentrations (g/L) were estimated using a refractometer (ATC 0-90, Akyol, Istanbul, Turkey) and calculated with the following equation [18]:

IgG (g/L) = −61.896 + 5.666 × Brix value(2)

All cows produced sufficient colostrum to adequately nourish their calves. Colostrum composition (total solids, fat, protein, casein, lactose, and urea) was analyzed using a milk analyzer (FT-120, Milkoscan, FOSS, Hillerød, Denmark). The milk analyzer was calibrated for raw bovine milk according to the manufacturer’s specifications and has not been independently validated for colostrum analysis in this study. However, FTIR-based analyzers have been widely used for colostrum composition analysis in previous research.

Calves were weighed using a livestock scale (Dikomsan HCW, Istanbul, Turkey) after they were expected to suckle their mothers and stand up following birth. Additionally, after weighing, body measurements including heart girth, withers height, and hip height were taken using a tape measure while calves were positioned on a flat surface. Body weight and body measurements were recorded weekly until weaning (day 63 after birth).

#### 2.3.3. Feed Analysis

During the study, 3–5 kg samples of corn silage were taken weekly and kept in an oven (KT 500, W.C. Heraeus, Hanau, Germany) at 60 °C for 48 h to determine the dry matter. Dry corn silage samples and other feeds (lucerne hay, wheat straw, dry period and lactation concentrates) from which 1–2 kg samples were taken at the beginning and end of the study were ground in a feed mill (ZM-200, Retsch, Haan, Germany) with a 1 mm sieve. Dry matter, crude protein, ether extract, and ash analyses were performed according to AOAC [19]. Neutral detergent fiber (NDF) and acid detergent fiber (ADF) were determined by the filter bag technique in a fiber analyzer (Ankom200 Fibre Analyzer, ANKOM Technology Corp., Macedon, NY, USA) using temperature-resistant α-amylase and sodium sulfite according to [20].

### 2.4. Statistical Analysis

Two cows were excluded prepartum due to adaptation issues, and one cow and its calf were excluded due to complications during calving. Thus, twenty-seven cows (Control: *n* = 13 and RUPCHOL: *n* = 14) and their calves were used in the experiment. The collected data were analyzed as prepartum (starting from the 21st day before calving until birth), calving (day 0), and postpartum (starting from the 1st day of lactation until the 29th day). Statistical analyses were performed in the SAS (version 9.4) package program [21,22]. Statistical analyses were based on repeated-measures data analysis, and the covariance structures used were Combined Symmetry (CS), First Order Autoregressive (AR(1)), First Order Autoregressive with Heterogeneous Variance Structures (ARH(1)), Unstructured, First Order Antedependence (ANTE(1)), and Distance Exponential (SP(POW)) [21,22]. The selection of the most appropriate covariance structure for the data was based on the smallest Akaike Information Criterion (AIC). The final covariance structures selected based on AIC were: First Order Autoregressive (AR(1)) for milk yield and DMI; Compound Symmetry (CS) for body weight and BCS; and Unstructured for blood metabolites. In analyses other than calving, the statistical model included treatment, week, and treatment × week interaction effect. In the statistical analysis of body weight at calving, a mixed model was used, treatment and lactation number were included as fixed effects, and animals within treatment groups were included as random effects in the model. Statistical differences were discussed as significant if *p* < 0.05 and as increasing or decreasing trend if 0.05 < *p* ≤ 0.10. The standard error of means in the tables was calculated as pooled means.

## 3. Results

### 3.1. Performance During Pre- and Postpartum

The prepartum period lasted 20.4 ± 1.52 (mean ± standard deviation) days in the control group and 21.4 ± 0.55 days in the RUPCHOL group. Gestation length, prepartum body weight, BCS, DMI, and rectal temperature are presented in Table 2. The prepartum body weight, BCS, and DMI of control and RUPCHOL-supplemented cows were not statistically different (*p* > 0.05 for each variable). The body weight and DMI of cows fed RUPCHOL were numerically lower than those in the Control group. The differences were 4.7% for body weight and 16.2% for DMI. On the other hand, feeding RUPCHOL decreased rectal temperature by 0.8 °C (38.4 °C vs. 39.2 °C and *p* = 0.042). There were changes in body weight (*p* < 0.01) and BCS (*p* = 0.043) of the groups depending on the weeks. Body weights and BCS of the control and RUPCHOL groups at calving were 623.1, 4.01 and 591, 3.94, respectively, and the values were statistically similar (*p* > 0.05 for all variables).

The effect of RUPCHOL supplementation on prepartum serum parameters is given in Table 3. Compared to control cows, serum AST (*p* = 0.054), NEFA (*p* = 0.069), and BHB (*p* = 0.089) levels tended to be lower in cows fed RUPCHOL. It was determined that there was no statistical difference between the groups in terms of glucose, Ca, Mg, P, total protein, BM, albumin, GGT, and cholesterol levels (*p* > 0.05 for all variables). On the other hand, the effect of the week on serum glucose levels was statistically significant (*p* = 0.034), but this effect was not significant for the other variables (*p* > 0.05 for all variables).

**Figure 1:**
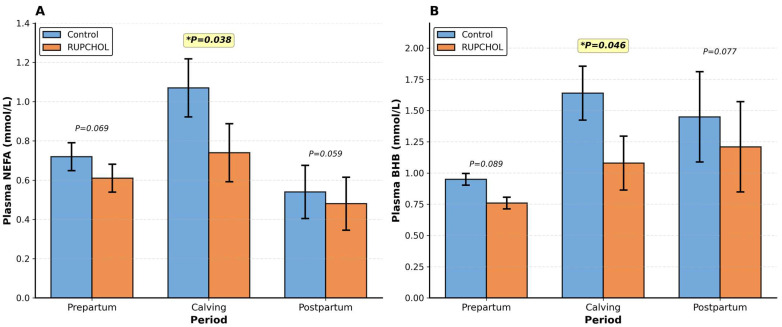
Effect of RUPCHOL supplementation on plasma metabolites during the peripartum period. (**A**) Plasma non-esterified fatty acid (NEFA) concentrations in control and RUPCHOL-supplemented cows across prepartum, calving, and postpartum periods. (**B**) Plasma β-hydroxybutyrate (BHB) concentrations in control and RUPCHOL-supplemented cows across prepartum, calving, and postpartum periods. Values represent least squares means ± SEM (pooled standard error of mean). * *p* < 0.05 indicates a significant difference between treatments.

Serum NEFA and BHB levels measured at calving were lower in RUPCHOL-fed cows compared to the control group. The reductions were 31% for NEFA (*p* = 0.038) and 34% for BHB (*p* = 0.046) (Table 3). Figure 1 provides a visual summary of these treatment differences at calving. Serum levels of glucose, Ca, Mg, P, total protein, BUN, albumin, GGT, and cholesterol measured at calving were statistically similar between groups (*p* > 0.05 for each variable). Animals in the RUPCHOL group also showed a tendency towards 17% lower serum AST levels at calving (*p* = 0.067).

Postpartum performance and rectal temperatures of the control and RUPCHOL groups are given in Table 4. Although body weight (560 kg vs. 539 kg), DMI (17.8 kg/day vs. 16.9 kg/day), and total milk yield (3888 kg vs. 3778 kg) were higher in cows fed RUPCHOL than in the control group, there was no statistical difference between the groups (*p* > 0.05 for each variable). On the other hand, BCS, milk yield, fat yield, protein yield, fat corrected milk yield, and rectal temperature of the cows in the control group were higher than those of the cows fed RUPCHOL, but there was no statistical difference between the groups (*p* > 0.05 for each variable). It was determined that BCS (*p* < 0.01), DMI (kg/day and % of BW, *p* < 0.01 for both variables) and milk yield (*p* < 0.01) and fat-corrected milk yield (*p* < 0.05) of the groups changed depending on the weeks; fat yield tended to change according to the week (*p* = 0.052), while body weight, protein yield, and rectal temperature were similar within weeks (*p* > 0.05 for each variable).

**Figure 2:**
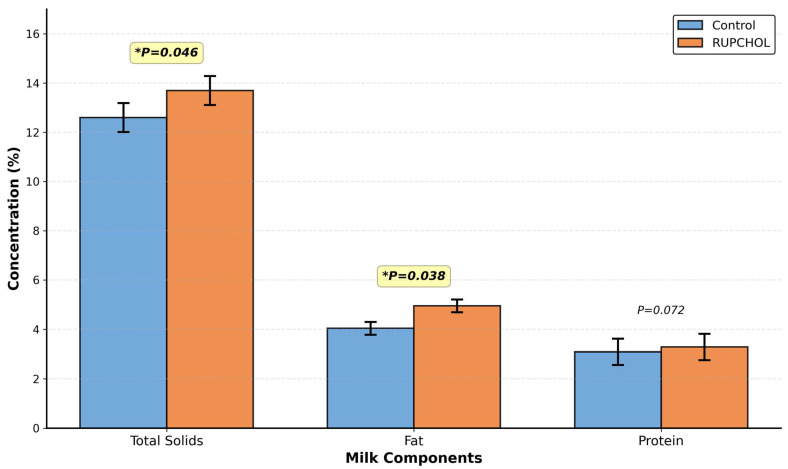
Effect of RUPCHOL supplementation on milk composition during the postpartum period. Concentrations of total solids, fat, and protein in milk from control and RUPCHOL-supplemented cows (days 7, 14, 21, and 28). Values represent least squares means ± SEM (pooled standard error of mean). * *p* < 0.05 indicates a significant difference between treatments.

Milk composition and somatic cell counts of the groups are given in Table 5. Lactose, casein, and urea-N levels of both control and RUPCHOL-fed cows were similar (*p* > 0.05 for each variable). On the other hand, it was found that milk total solids (*p* = 0.046) and fat (*p* = 0.038) levels were higher in RUPCHOL-fed cows compared to the control group, while milk protein level tended to be higher (*p* = 0.072). These differences in milk total solids, fat, and protein concentrations are visually summarized in Figure 2. There was no statistical difference between the groups in terms of somatic cell count (*p* > 0.05). It was determined that somatic cell count showed a change depending on the week (*p* = 0.043).

Postpartum serum parameters are given in Table 6. Postpartum serum levels of glucose, Ca, Mg, P, total protein, BUN, albumin, AST, GGT, and cholesterol were similar in control and RUPCHOL-fed cows (*p* > 0.05 for each variable). Serum NEFA and BHB levels were 11% (*p* = 0.059) and 17% (*p* = 0.077) lower, respectively, in RUPCHOL-fed cows compared to the control group. It was determined that the week was effective on serum glucose (*p* < 0.01), Ca (*p* = 0.016), BUN (*p* < 0.01), AST (*p* < 0.01), GGT (*p* = 0.013), NEFA (*p* = 0.043), and BHB (*p* < 0.01) levels, but not on Mg, P, total protein, albumin, and cholesterol levels (*p* > 0.05 for each variable).

### 3.2. Partum

The effects of RUPCHOL supplementation to the maternal ration in the prepartum period on the birth characteristics of calves are given in Table 7. Birth weight of calves did not differ between groups (*p* > 0.05), and the average birth weight was 32 kg. Prenatal RUPCHOL supplementation did not affect heart girth, withers height, hip height, and body length at calving (*p* > 0.05 for each variable). Lactation number tended to affect the birth weight of calves (*p* = 0.067). Calf birth weights of cows in the 1st, 2nd, and 3rd lactation were 26.4 ± 2.26 (least squares mean ± standard error), 32.3 ± 1.22, and 37.1 ± 1.92, respectively. The variables analyzed in female and male calves were statistically similar (*p* > 0.05 for each variable).

### 3.3. Effect on Colostrum IgG and Nutrient Composition

The effects of RUPCHOL supplementation of maternal diets on colostrum IgG and nutrient levels are given in Table 8. There was no statistical difference (*p* > 0.05) for colostrum IgG levels between groups. In addition, colostrum total solids, fat, protein, casein, lactose, and urea-N were statistically similar (*p* > 0.05 for each variable). It was found that colostrum variables, except fat (*p* > 0.05) and lactose (*p* > 0.05), were affected by sampling time (0 h and 36 h) (*p* < 0.01 for each variable), and the T × Hour interaction was not statistically significant (*p* > 0.05 for each variable).

### 3.4. Impact on Performance from Birth to Weaning

The effects of RUPCHOL supplementation to maternal diets in prepartum on performance from birth to weaning are given in Table 9. It was found that the body weight, heart girth, withers height, hip height, and body length of calves born from RUPCHOL-fed dams and calves born from control group dams were statistically similar (*p* > 0.05 for each variable) between birth and the 35th day. Body weight, heart girth, withers height, hip height, and body length varied according to week (*p* < 0.05 for each variable). The effect of week tended to be significant (*p* = 0.091) on withers height. The T × Week interaction affected body weights (*p* < 0.05). Between birth and the 35th day, male calves were 20% heavier than female calves (*p* < 0.05).

It was determined that the performance of calves born from RUPCHOL-fed dams and calves born from control group dams was similar (*p* > 0.05 for each variable) between the 36th and 63rd days. It was determined that heart girth (*p* < 0.01) and body length (*p* < 0.01) of the calves changed significantly depending on the week. In addition, it was also determined that the effect of week on body weight, withers, and hip height was not significant (*p* > 0.05 for each variable). The T × Week interaction was not statistically significant (*p* > 0.05 for each variable) in relation to the variables analyzed. Male calves were approximately 16% heavier than female calves (Sex effect, *p* = 0.028). For the other variables, the effect of sex was not statistically significant (*p* > 0.05).

The treatments did not affect the weaning performance variables (*p* > 0.05 for each variable). In terms of live weight at weaning, male calves were approximately 9% heavier than female calves (*p* < 0.05). On the other hand, it was also found that heart girth, withers height, hip height, and body length were similar in male and female calves (*p* > 0.05 for each variable).

## 4. Discussion

### 4.1. Performance Parameters

In the present study, the use of RUPCHOL during the peripartum period did not affect body weight, BCS, DMI, milk yield, and somatic cell count. The fact that there was no difference in the DMI of the groups was also considered to be effective because the body weight and BCS were similar. In the literature, there are studies [12,23,24] that reported that live weight, BCS, and DMI were statistically similar in cows with and without choline supplementation during the transition period. On the other hand, there are also studies [25] that reported an increase in DMI but no change in body weight and BCS in cows supplemented with choline. The differences between studies may be related to animal characteristics, such as body weight or lactation number. Feeding systems, including TMR versus separate roughage and concentrate feeding, may also contribute.

Rumen-protected choline supplementation did not affect total milk yield up to day 150 of lactation. The effect of RUPCHOL supplementation on milk yield of peripartum cows varies [26,27]. Indeed, Refs. [12,28,29] reported that RUPCHOL increased milk yield, while Refs. [25,30] reported no change in milk yield. In these studies, the duration of RUPCHOL supplementation varied. This variation may partly explain the inconsistent effects observed on milk yield. For example, in some studies [23,31], RUPCHOL supplementation started from prepartum and continued until postpartum days 60 to 80, while in another study [32], it was continued until postpartum day 21. On the other hand, Ref. [24] reported that the supplementation affected yield during the transition period and that rumen-protected choline supplementation after postpartum day 22 was not beneficial. Ref. [33] explained this effect by the fact that the effect of rumen-protected choline on increasing the circulating choline biomolecule concentration was higher during the transition period. Furthermore, Ref. [7] suggested that the response observed in milk yield, related to RUPCHOL supplementation when methionine is low, may not be observed when methionine is high.

In the present study, the methionine requirement estimated using the NASEM [15] Dairy Cow Software (V8 R2024.05.05) was approximately 41 g/day per cow during the postpartum period, based on an average dry matter intake of approximately 17 kg/day and an expected milk yield of 27 kg/day. This value represents the total daily methionine intake. The calculated methionine supply appeared to be adequate for cows producing 25–30 kg of milk per day, which may explain why RUPCHOL supplementation did not further enhance milk yield in this study. At this point, it should be noted that several studies [28,34,35] have reported variable responses to methionine supplementation in dairy cows. The differences in outcomes may be associated with the physiological status of the animals, the basal diet composition, and the duration of supplementation. In our study, methionine requirements during the postpartum period appeared to be sufficiently met through the basal diet, resulting in no additional milk yield response to RUPCHOL supplementation, even though improvements in metabolic parameters were observed.

The addition of rumen-protected choline decreased rectal temperature in prepartum. Phosphatidylcholine, which contains choline in its structure, also plays a role in maintaining intestinal villus integrity [36]. Preventing damage to the gastrointestinal barrier may prevent bacteria or toxins from entering the bloodstream and inflammatory responses [37]. Furthermore, choline plays a role in modulating the immune response [38,39] and may alleviate inflammation [32]. Thus, the rise in body temperature will also be prevented.

Recent studies have further demonstrated the importance of immune modulation and antimicrobial strategies in maintaining health during metabolically challenging periods in livestock [6,40]. Furthermore, dietary fatty acid supplementation, including EPA and DHA, has been shown to modulate lipid metabolism pathways in various species [41], which may share mechanistic similarities with choline’s lipotropic effects.

The addition of RUPCHOL increased milk total solids, fat, and protein levels. In the literature, there are studies [12,25,26] that reported that rumen-protected choline supplementation increased milk protein concentration. Acting as a methyl donor, rumen-protected choline may promote the conservation of methionine, its transport to the mammary tissue in greater amounts, and its utilization in protein synthesis [11,27,42]. The increase in milk fat level with the addition of rumen-protected choline may be attributed to the positive effect on the transport of fatty acids mobilized from adipose tissue to the mammary tissue via the liver during the peripartum period [27]. Thus, the availability of lipids for milk fat synthesis increases, and the milk fat level may also increase. On the other hand, there are also studies in the literature [28,30] reporting that rumen-protected choline supplementation does not affect milk fat level. However, the digestibility of organic matter (including ether extract) was increased in lactating cows fed 15 or 30 g/day rumen-protected choline compared to non-fed cows [43]. Lactating rats fed choline-deficient diets were reported to have shorter jejunum villi and lower fat absorption ability than those supplemented with choline or lecithin [36]. The lack of effect of RUPCHOL on milk lactose concentration in the present study is consistent with the lack of increase in milk yield. Furthermore, as stated in the metabolic parameters section of the present work, the serum glucose concentrations of the groups were similar. As it is known, lactose is synthesized from one molecule of glucose and galactose, and milk lactose concentration constitutes the bulk portion of milk [44]. Therefore, the increase in milk dry matter in the present study was due to the increase in fat and protein levels. However, the absence of an isoenergetic placebo diet should be considered when interpreting changes in milk fat and total solids, as differences in energy supply cannot be fully excluded.

### 4.2. Metabolic Parameters

The addition of RUPCHOL did not affect serum glucose, Ca, Mg, P, total protein, BUN, albumin, and GGT concentrations during the experiment. The literature reporting the effect of rumen-protected choline supplementation on these metabolite concentrations is variable. Recent studies (e.g., Refs. [24,33]) reported that choline supplementation during the transition period increased serum total Ca concentrations only between calving and postpartum d 3, resulting in fewer cases of subclinical hypocalcemia. In the present study, Ca levels measured in blood samples of cows supplemented with RUPCHOL were numerically higher than those of the control group, although not statistically significant (prepartum: control group 8.51 mg/dL and RUPCHOL group 8.93 mg/dL, calving: control group 7.12 mg/dL and RUPCHOL group 7.83 mg/dL, postpartum: control group 8.78 mg/dL and RUPCHOL group 9.16 mg/dL).

Serum cholesterol concentrations of the control and RUPCHOL-supplemented groups were similar during the study. Most of the cholesterol in the body is synthesized by the liver, small intestinal epithelium, and adipose tissue using mainly acetate [44]. Ref. [4] attributed the positive association between rumen-protected choline intake and serum cholesterol levels to the ability of rumen-protected choline to reduce hepatic triacylglycerol concentrations. In other words, choline increases the synthesis and export of lipoproteins by the small intestine (chylomicrons) and liver (very-low-density lipoproteins), leading to increased blood total cholesterol concentrations. However, the literature reporting the effect of rumen-protected choline supplementation on blood cholesterol concentrations in early lactation dairy cows, i.e., under conditions of negative energy balance, varies. Indeed, Refs. [45,46] reported an increase, and Ref. [47] reported a decrease, while [12,28,30,34,48] reported no effect. This discrepancy between studies on choline and blood cholesterol may be attributed to the time of blood sampling, milk yield, and DMI levels of the cows in the study, and the severity of negative energy balance.

In the present study, RUPCHOL supplementation during the transition period resulted in a decrease in serum NEFA and BHB concentrations. Similarly, Ref. [49] reported a decrease in NEFAs with choline supplementation, which resulted in a decrease in hepatic fat accumulation. However, several studies in the literature have reported varying effects of different rumen-protected choline products on serum BHB and NEFA concentrations. Several studies reported no significant changes in BHB concentrations with rumen-protected choline supplementation [27,28,29], whereas others observed increased BHB levels in cows supplemented with specific rumen-protected choline products [24,33]. It is important to note that these studies employed different formulations of rumen-protected choline; therefore, direct comparisons should be made cautiously, as not all products have the same rumen protection efficiency or bioavailability. No differences were found in post-calving serum glucose and fatty acid concentrations between control and supplemented lactating cows [25,30]. Conversely, decreases in serum BHB and fatty acid concentrations, as well as increases in serum glucose, were reported following supplementation [12,32].

### 4.3. Calves Performance

In the present study, body weight and body measurements (heart girth, withers height, hip height, and body length) measured at birth and from birth to weaning were used to compare the performance of calves born to dams with and without RUPCHOL supplementation. RUPCHOL supplementation did not affect the aforementioned variables of the groups. Ref. [50] reported that feeding rumen-protected choline during late gestation improved colostrum IgG gross absorption, milk replacer and starter concentrate consumption, and reduced inflammatory responses in the postnatal period. However, Ref. [51] reported that calves receiving 20.4 g/day, 13.6 g/day, and 0 g/day of choline ion from prepartum day 24 did not differ in birth and first 3-week body weights. In addition, there was a difference between choline ion doses in terms of daily body weight gain, but no difference was found between these groups and the control group [51]. Ref. [52] did not find any effect of feeding rumen-protected choline in prepartum on calf birth weight and 8th week body weight. In the present study, although not statistically significant, a numerical difference of 7% was found between the birth weights of the groups. The differences between the studies can be attributed to several factors, including timing and duration of choline supplementation, provision of alternative methyl convertors (e.g., methionine or betaine), ration nutrient composition, and maternal characteristics (e.g., number of lactations, Ref. [52]).

Regarding the timing of choline supplementation, it has been reported that choline supplementation to bovine embryos during the preimplantation period increases calf birth weight, weaning weight, and body weight–hip height ratio, and leads to postnatal changes in muscle DNA methylation, which is also associated with genes related to anabolic process and cellular growth [53]. On the other hand, Ref. [51] reported that prepartum maternal variables (e.g., BCS, oxidant status, metabolism, etc.) can affect calf performance. Therefore, it could be argued that the effects of prenatal rumen-protected choline may depend on the prepartum metabolic balance of the dam prior to treatment. It has also been reported that in the case of nutrient intake disruptions, nutrients from the dam are primarily directed to vital organs such as the brain, liver, and heart [54]. Therefore, there may be no change in body weight and body measurements until birth or weaning, and the effect may be seen later in life. Indeed, Ref. [4] reported that the improvement in growth and feed evaluation efficiency was observed at 50 weeks of age.

### 4.4. Colostrum IgG and Its Chemical Composition

Supplementation of RUPCHOL to the maternal ration did not affect IgG and nutrient composition in colostrum samples taken at birth and 36 h after birth. Goats given 5 and 10 g/day of rumen-protected choline from kidding to weaning had higher milk fat and protein levels and lower lactose levels than controls and goats given 2.5 g/day rumen protected choline [55]. It was evaluated that the differences between the studies could be due to the species (goats versus cattle and kids receiving their mother’s milk during suckling, while calves received tank milk) and the duration of rumen-protected choline use (prepartum versus mating to weaning). Furthermore, Ref. [50] reported that colostrum fat, total protein, lactose, and somatic cell counts were similar in cows with and without rumen-protected choline supplementation in the prepartum period. However, calves fed colostrum from cows fed diets supplemented with rumen-protected choline had higher serum IgG concentrations than control calves, and it was suggested that this effect may be due to the formation of different choline metabolites or concentrations in colostrum by rumen-protected choline feeding [50,51]. However, it should be noted that the FTIR analyzer used in the present study was calibrated for raw bovine milk and has not been independently validated for colostrum analysis. Nevertheless, infrared-based spectroscopic approaches, including FTIR and FT-NIR techniques, have previously been applied for the estimation of major colostral components in bovine colostrum [56,57]. Therefore, while the present results should be interpreted with caution, the use of FTIR-based predictions for colostrum composition is supported by previous methodological studies.

## 5. Conclusions

Rumen-protected choline supplementation (15 g choline chloride/day) from 21 days prepartum to 28 days postpartum improved metabolic indicators and milk composition in peripartum dairy cows. These effects were most evident around the time of calving. Specifically, RUPCHOL reduced plasma NEFA concentrations at calving (*p* = 0.038) and plasma BHB concentrations at calving (*p* = 0.046), indicating improved metabolic status during the critical transition period. Additionally, RUPCHOL increased milk total solids (*p* = 0.046) and fat (*p* = 0.038) content, with protein tending to increase (*p* = 0.072), suggesting enhanced milk quality. However, RUPCHOL did not affect total milk yield, colostrum IgG content, or calf growth from birth to weaning. Study limitations include the relatively small sample size (*n* = 27) and moderate milk production level (25–30 kg/day), which may have limited the detection of treatment effects. Additionally, the lack of an isoenergetic placebo control should be considered when interpreting milk fat results. Future studies with larger sample sizes, higher-producing cows, and appropriate placebo controls are warranted to confirm these findings and explore potential long-term effects on calf performance.

## Figures and Tables

**Table 1:** Ingredient and chemical composition of pre- and postpartum TMR.

Ingredient, % of DM	Prepartum	Postpartum
Wheat straw	27.78	
Corn silage	47.05	25.84
Lucerne hay		17.81
Ground barley grain	3.94	17.39
Corn grain		7.61
Soybean meal		7.79
Sunflower meal	8.97	
Canola meal		6.82
Wheat bran	4.32	6.60
Corn bran	4.27	
Whole cotton seed		6.82
Fat, safflower oil	1.76	
Molasses	0.87	
Limestone	0.18	0.37
Salt	0.18	0.49
Sodium bicarbonate		0.62
Mineral—vitamin premix	0.08 ^1^	0.61 ^2^
Ammonium chloride	0.62	
Nutrient composition (% diet DM, unless noted)		
DM, % as fed	52.0	58.3
Crude protein	10.9	18.3
NDF	47.4	31.2
ADF	29.9	19.2
Ether extract	4.4	5.5
Ash	6.3	7.2
NEL, Mcal/kg ^3^	1.64	1.79
DCAD, mEq/kg ^3^	25	237
Methionine (g/d) ^3^	21	41

^1^ Prepartum mineral—vitamin premix: 2.5 g/kg manganese, 1.5 g/kg iron, 0.5 g/kg copper, 2.5 g/kg zinc, 0.0125 g/kg cobalt, 0.075 g/kg iodine, 0.03 g/kg selenium, 500,000 IU/kg vitamin A, 100,000 vitamin D_3_, E 25 g/kg vitamin, 7.5 g/kg beta-carotene, and 30 g/kg niacin. ^2^ Postpartum mineral—vitamin premix: 50 g/kg manganese, 50 g/kg iron, 10 g/kg copper, 50 g/kg zinc, 0.15 g/kg cobalt, 0.8 g/kg iodine, 0.15 g/kg selenium, 15,000,000 IU/kg vitamin A, 3,000,000 vitamin D_3_, 20 g/kg vitamin E, and 150 g/kg niacinamide. ^3^ Net energy lactation (NEL) and dietary cation–anion diference (DCAD) were estimated using NASEM [15] Dairy Cattle Nutrition software (V8 R2024.05.05).

**Table 2:** Effect of RUPCHOL supplementation during the peripartum period on prepartum and calving performance of transition cows.

Item	Treatment (T) ^1^		SEM ^2^	*p*-Value		
	Control	RUPCHOL		T	Week	T × Week
Prepartum						
Gestation length (d)	277	281	2.53	0.962	-	-
Body weight (kg)	667.2	635.9	37.47	0.588	<0.01	0.752
BCS ^3^	3.83	3.79	0.254	0.913	0.043	0.706
DMI ^4^ (kg/day)	13.6	11.4	0.770	0.119	0.200	0.719
DMI ^4^ (% Body weight)	2.05	1.80	0.115	0.202	0.101	0.743
Rectal temperature (°C)	39.2	38.4	0.21	0.042	0.558	0.906
At calving ^5^						
Body weight (kg)	623.1	591.0	32.31	0.521	-	-
BCS ^3^	4.01	3.94	0.436	0.768	-	-

^1^ RUPCHOL—rumen-protected choline, ^2^ SEM—standard error of mean, ^3^ BCS—body condition score, ^4^ DMI—dry matter intake, ^5^ Only treatment effect was included in the statistical model.

**Table 3:** Effect of RUPCHOL supplementation during the peripartum period on serum parameters in prepartum and calving.

	Treatment (T) ^1^		SEM ^2^	*p*-Value		
Item	Control	RUPCHOL		T	Week	T × Week
Prepartum						
Glucose (mg/dL)	72.6	69.1	3.34	0.846	0.034	0.704
Ca (mg/dL)	8.51	8.93	0.225	0.219	0.885	0.457
Mg (mg/dL)	2.03	1.87	0.147	0.473	0.107	0.399
P (mg/dL)	5.23	4.63	0.827	0.345	0.748	0.814
Total protein (g/dL)	7.32	7.30	0.160	0.968	0.290	0.435
BUN ^3^ (mg/dL)	15.5	10.6	2.92	0.298	0.185	0.327
Albumin (g/dL)	3.43	3.13	0.106	0.102	0.584	0.343
AST ^3^ (IU/L)	109.5	73.6	9.38	0.054	0.127	0.809
GGT ^3^ (IU/L)	17.7	22.9	2.81	0.267	0.011	0.646
Cholesterol (mg/dL)	113.7	90.9	18.93	0.443	0.380	0.382
NEFA ^3^ (mmol/L)	0.72	0.61	0.071	0.069	0.410	0.843
BHB ^3^ (mmol/L)	0.95	0.76	0.047	0.089	0.397	0.749
At calving ^4^						
Glucose (mg/dL)	74.9	71.3	6.17	0.751	-	-
Ca (mg/dL)	7.12	7.83	0.348	0.843	-	-
Mg (mg/dL)	2.42	2.69	0.461	0.643	-	-
P (mg/dL)	5.76	5.97	0.764	0.816	-	-
Total protein (g/dL)	7.04	7.35	0.654	0.457	-	-
BUN ^3^ (mg/dL)	13.2	14.0	1.29	0.736	-	-
Albumin (g/dL)	3.85	3.78	0.301	0.403	-	-
AST ^3^ (IU/L)	127.3	106.1	6.49	0.067	-	-
GGT ^3^ (UI/L)	19.3	20.6	1.89	0.434	-	-
Cholesterol (mg/dL)	118.9	115.6	8.96	0.835	-	-
NEFAs ^3^ (mmol/L)	1.07	0.74	0.148	0.038	-	-
BHB ^3^ (mmol/L)	1.64	1.08	0.216	0.046	-	-

^1^ RUPCHOL—rumen-protected choline, ^2^ SEM—standard error of mean, ^3^ BUN—blood urea nitrogen, AST—aspartate aminotransferase, GGT—gamma-glutamyl transferase, NEFAs—non-esterified fatty acids, BHB—beta-hydroxybutyric acid, ^4^ Only treatment effect was included in the statistical model.

**Table 4:** Effect of RUPCHOL supplementation during the peripartum period on postpartum performance.

	Treatment (T) ^1^			*p*-Value		
Item	Control	RUPCHOL	SEM ^2^	T	Week	T × Week
Body weight (kg)	538.8	560.3	26.04	0.571	0.940	0.805
BCS ^3^	3.34	3.07	0.563	0.578	<0.01	0.782
DMI ^4^ (kg/d)	16.9	17.8	1.07	0.540	<0.01	0.461
DMI ^4^ (% body weight)	3.15	3.43	0.198	0.364	<0.01	0.831
Milk yield (kg/d)	27.5	26.4	1.97	0.696	<0.01	0.798
Fat yield (kg/d)	1.09	1.27	0.089	0.776	0.052	0.862
Protein yield (kg/d)	0.79	0.88	0.114	0.614	0.161	0.275
Fat-corrected milk	25.8	23.7	2.74	0.214	0.013	0.447
Total milk yield ^5^ (kg)	3778.3	3888.2	321.3	0.826	-	-
Rectal temperature (°C)	38.8	38.4	0.87	0.788	0.467	0.647

^1^ RUPCHOL—rumen-protected choline, ^2^ SEM—standard error of mean, ^3^ BCS—body condition score, ^4^ DMI—dry matter intake, ^5^ Total milk yield up to day 150 of lactation. Only the treatment effect was included in the statistical model.

**Table 5:** Effect of RUPCHOL supplementation during peripartum on milk composition and somatic cell count.

	Treatment (T) ^1^			*p*-Value		
Item	Control	RUPCHOL	SEM ^2^	T	Week	T × Week
Total solids (%)	12.6	13.7	0.59	0.046	0.138	0.798
Fat (%)	4.04	4.95	0.26	0.038	0.193	0.699
Protein (%)	3.09	3.29	0.53	0.072	0.151	0.557
Lactose (%)	4.71	4.66	0.15	0.839	0.189	0.311
Casein (%)	2.52	2.69	0.16	0.514	0.213	0.251
Urea-N (mg/dL)	20.1	17.3	1.24	0.192	0.782	0.265
Somatic cell count (×10^3^ cell/mL)	167.8	155.1	0.174	0.465	0.043	0.850

^1^ RUPCHOL—rumen-protected choline, ^2^ SEM—standard error of mean.

**Table 6:** The effect of RUPCHOL supplementation during peripartum on postpartum serum parameters.

	Treatment (T) ^1^			*p*-Value		
Item	Control	RUPCHOL	SEM ^2^	T	Week	T × Week
Glucose (mg/dL)	62.7	63.4	1.87	0.784	<0.01	0.681
Ca (mg/dL)	8.78	9.16	2.20	0.386	0.016	0.913
Mg (mg/dL)	2.71	2.83	0.596	0.603	0.864	0.738
P (mg/dL)	5.01	5.54	0.967	0.836	0.794	0.603
Total protein (g/dL)	7.88	6.89	0.945	0.543	0.631	0.709
BUN ^3^ (mg/dL)	16.4	15.3	1.57	0.327	<0.01	0.506
Albumin (g/dL)	3.77	4.03	0.414	0.418	0.371	0.573
AST ^3^ (IU/L)	135.7	120.3	33.29	0.743	<0.01	0.861
GGT ^3^ (UI/L)	26.41	27.45	6.61	0.831	0.013	0.456
Cholesterol (mg/dL)	125.6	121.8	31.40	0.703	0.678	0.640
NEFAs ^3^ (mmol/L)	0.54	0.48	0.135	0.059	0.043	0.749
BHB ^3^ (mmol/L)	1.45	1.21	0.362	0.077	<0.01	0.675

^1^ RUPCHOL—rumen-protected choline, ^2^ SEM—standard error of mean, ^3^ BUN—blood urea nitrogen, AST—aspartate aminotransferase, GGT—gamma-glutamyl transferase, NEFAs—non-esterified fatty acids, BHB—beta-hydroxybutyric acid.

**Table 7:** The effect of RUPCHOL supplementation of maternal ration on gestation length and birth characteristics of calves.

	Treatment (T) ^1^			*p*-Value		
Item	Control	RUPCHOL	SEM ^2^	T	Lactation Number	Sex
Birth weight (kg)	30.8	33.1	1.29	0.273	0.067	0.349
Heart girth (cm)	72.0	71.7	1.10	0.873	0.425	0.995
Withers height (cm)	71.2	70.7	1.68	0.834	0.984	0.346
Hip height (cm)	73.6	75.9	2.88	0.615	0.588	0.384
Body length (cm)	44.4	45.2	1.58	0.377	0.363	0.675

^1^ RUPCHOL—rumen-protected choline, ^2^ SEM—standard error of mean.

**Table 8:** Effect of RUPCHOL supplementation to maternal ration on colostrum IgG and nutrient composition.

	Treatment (T) ^1^			*p*-Value		
Item	Control	RUPCHOL	SEM ^2^	T	Hour	T × Hour
IgG ^3^ (g/L)	61.5	62.2	9.65	0.966	<0.01	0.798
Total solids (%)	21.0	22.3	1.28	0.480	<0.01	0.355
Fat (%)	2.89	2.93	0.81	0.974	0.649	0.795
Protein (%)	12.6	14.4	1.39	0.406	<0.01	0.410
Casein (%)	8.59	9.52	0.90	0.493	<0.01	0.792
Lactose (%)	3.31	3.04	0.43	0.669	0.500	0.597
Urea-N (mg/dL)	43.1	51.7	7.48	0.448	<0.01	0.526

^1^ RUPCHOL—rumen-protected choline, ^2^ SEM—standard error of mean, ^3^ IgG—Immunoglobulin G.

**Table 9:** Effect of RUPCHOL supplementation of maternal ration on the performance of calves from birth to weaning.

	Treatment (T) ^1^					*p*-Value			
	Control		RUPCHOL						
Item	Female	Male	Female	Male	SEM ^2^	T	Week	T × Week	Sex
*n*	8	5	6	8					
Between calving and d 35									
Body weight (kg)	30.8	41.6	37.7	40.6	2.17	0.237	<0.01	0.020	0.022
Heart girth (cm)	74.4	79.1	78.7	79.4	1.38	0.153	<0.01	0.567	0.112
Withers height (cm)	72.9	76.1	73.9	76.2	1.57	0.756	0.091	0.692	0.144
Hip height (cm)	78.1	80.2	78.2	80.8	1.78	0.862	<0.01	0.574	0.249
Body length (cm)	45.0	46.2	45.9	46.5	1.01	0.814	<0.01	0.117	0.298
Between d 36 and d 63									
Body weight (kg)	43.0	56.3	52.0	54.3	2.63	0.245	0.019	0.212	0.028
Heart girth (cm)	85.8	87.8	86.1	87.5	1.59	0.268	<0.01	0.998	0.661
Withers height (cm)	79.6	81.7	79.4	80.9	1.89	0.781	0.021	0.750	0.419
Hip height (cm)	86.6	87.2	84.3	86.5	1.79	0.450	0.025	0.306	0.470
Body length (cm)	48.9	49.9	49.3	49.4	1.14	0.927	<0.01	0.128	0.653
At weaning ^3^ (d 63)									
Body weight (kg)	59.1	66.8	62.1	65.0	2.04	0.349	-	-	0.039
Heart girth (cm)	90.7	96.3	90.4	92.3	1.43	0.790	-	-	0.490
Withers height (cm)	83.0	86.0	83.8	85.0	1.78	0.703	-	-	0.374
Hip height (cm)	87.3	89.5	88.7	90.0	1.98	0.801	-	-	0.845
Body length (cm)	52.3	54.7	50.0	50.8	1.83	0.587	-	-	0.379

^1^ RUPCHOL—rumen-protected choline, ^2^ SEM—standard error of mean. ^3^ Only treatment and sex effects were included in the statistical model.

## Data Availability

The data that support the findings of this study are available upon reasonable request from the corresponding author (Ugur Serbester).

## Abbreviations

The following abbreviations are used in this manuscript:

RUPCHOL	Rumen-protected choline
DMI	Dry matter intake
FCM	Fat-corrected milk
NEFAs	Non-esterified fatty acids
BHB	β-hydroxybutyric acid
IgG	Immunoglobulin G
BUN	Blood urea nitrogen
AST	Aspartate aminotransferase
GGT	Gamma-glutamyl transferase
SEM	Standard error of mean
